# Surface Modification of TiO_2_ Nanorods for Dye Removal: Photodegradation vs Adsorption Activity

**DOI:** 10.1021/acsomega.5c02952

**Published:** 2025-11-03

**Authors:** Chiara Lo Porto, Daniele Conelli, Carlo Nazareno Dibenedetto, Marinella Striccoli, Fabio Palumbo, Roberto Grisorio, Gian Paolo Suranna

**Affiliations:** † Dipartimento di Ingegneria Civile, Ambientale, del Territorio, Edile e di Chimica (DICATECh), 18951 Politecnico di Bari, Via Orabona 4, 70125 Bari, Italy; ‡ CNR IPCF − Istituto per i Processi Chimico Fisici, UOS Bari, Via Orabona 4, 70126 Bari, Italy; § CNR-NANOTEC − Institute of Nanotechnology, UOS Bari, Via Orabona 4, 70126 Bari, Italy; ∥ CNR-NANOTEC − Institute of Nanotechnology, c/o Campus Ecoteckne, Via Monteroni, 73100 Lecce, Italy

## Abstract

Colloidal titanium dioxide nanoparticles (NPs) represent a versatile class of semiconductors due to their tunable morphologies and organic shell formulations via the tools offered by synthetic nanochemistry. While largely exploited to remove industrial pigments from wastewater, scarce attention has been thus far devoted to modulating and rationalizing the different capacities of TiO_2_-based NPs in photodegradation and adsorption processes, posing the different dye structures in relation to the surface chemistry and morphology of the relevant semiconductor. This study aims at effectively understanding the potentialities and limits of surface modification in TiO_2_ nanorods (NRs) for dye removal from wastewater by adsorbing or photodegradation pathways. The NRs are initially synthesized with oleic acid (OA) as capping ligands (NR-OA) and subsequently functionalized with catechol (Cat) to generate the surface-modified analogue (NR-Cat) preserving the original aspect ratio of the inorganic core. Notwithstanding the partial replacement of the pristine capping ligands (supported by TGA and XPS analyses), an evident reduction of the band gap in NR-Cat (1.72 eV) in comparison to NR-OA (2.75 eV) with positive implications in its light-harvesting properties is observed. However, NR-Cat demonstrates to be highly efficient in water remediation solely by adsorption processes and only for cationic colorants, such as methylene blue and rhodamine B. This behavior is attributed to the increasingly negative surface charge of NR-Cat promoted by the surface modification, as proven by ζ-potential measurements. At the same time, the photoactivity of NR-Cat in dye removal is heavily affected by the sole activation of hydroxyl radicals for the degradation process, as suggested by the position of the material conduction band (hampering the formation of reactive oxygen species) and confirmed by scavenging experiments. Conversely, the photoinduced processes are revealed to be the preferential pathway for dye removal in the case of NR-OA, acting only on xanthene-based (eosin B, rhodamine B, Rose Bengal) and methylene blue colorants. Therefore, this study demonstrates that applying a systematic NP/dye approach could be a promising strategy for unlocking deeper insights into the fundamental behavior of semiconductors during adsorption and photocatalytic pathways, thus paving the way to innovative nanomaterials designed for mitigating the environmental problems raised by wastewaters.

## Graphical abstract

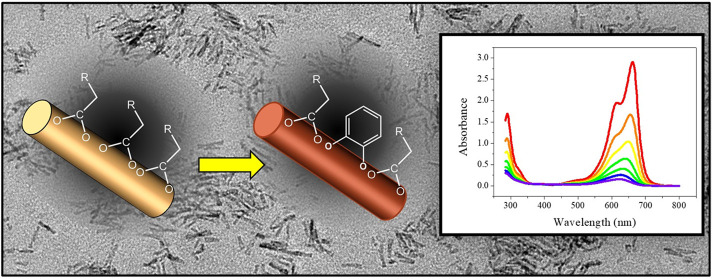

## Introduction

In wastewater treatment of textile industries, photocatalysis is a widely employed approach for the degradation of organic pigments^1,2^ that can pose a threat to human health and environment.^3–5^ The photocatalytic processes utilize semiconducting materials to capture light energy for either the direct or the indirect generation of active species (*i.e*., holes, electrons, reactive oxygen species) capable of degrading an organic pollutant into harmless substances. For the same environmental problem, adsorption for removing dyes can be considered an equivalently efficient and sustainable method. The choice of the semiconducting material privileges its light-harvesting properties or porosity in relation to the implementation of photocatalytic or adsorption strategies, respectively. A wide range of semiconductors has been engineered to adsorb organic contaminants from aqueous solutions, among which several metal oxides have been explored.^6^ Generally, removal efficiency by adsorption is strictly correlated to the material surface area favoring dye/semiconductor contact from a mere quantitative point of view.^7,8^ Conversely, scarce attention has been focused on the refinement of the surface chemistry of the semiconductor to maximize the removal yields by adsorption.

Titanium dioxide (TiO_2_) in the form of nanoparticles (NPs) stands as one of the most extensively studied inorganic materials due to its manifold properties, promoting the flourishing of applications in photocatalysis,^9,10^ energy conversion,^11^ biomedical technologies,^12–14^ and environmental remediation.^15^ The main issue of TiO_2_ NPs resides in its high band gap (3.03 eV for rutile and 3.20 eV for anatase)^16^ restricting their photoinduced activation to the sole UV radiation with evident limitations for real applications of this material in photocatalysis.^17^

Generally, two conceptual schemes in designing TiO_2_ NPs are followed to attenuate this drawback. The first one intervenes on their shape with the tools of colloidal synthesis: an elongated form, in fact, tends to hinder the hole/electron couple recombination and maximizing the intrinsic number of photoinduced charges for absorbed photon.^18,19^ The second one relies on the surface modification of TiO_2_ NPs with a sensitizer aiming at enhancing their absorption properties.^20–23^ The binding of the ligand on the TiO_2_ surface induces a photogenerated charge transfer that can follow a one-step (type I) or a two-step (type II) pathway. In type I sensitized systems, the photoexcitation takes place within the sensitizer, followed by charge transfer to the TiO_2_ conduction band (CB). In type II systems, a much faster photoinduced electron transfer occurs directly from the sensitizer to the TiO_2_ CB.^24^ While several articles have investigated dye removal with TiO_2_-based NPs,^25–27^ to the best of our knowledge no one has explicitly explored the intricacies of adsorption and photodegradation pathways of differently structured colorants as a function of the semiconductor’s surface chemistry and morphology.

In this study, we merge the two approaches to construct differently functionalized TiO_2_ nanorods (NRs) and compare their photocatalytic and adsorption performances with the conventional quasi-spherical P25 benchmark. The functionalization of the pristine NR capped with oleic acid (NR-OA) was carried out with catechol to form a mixed organic shell analogue (NR-Cat), which clearly exhibits a marked absorption in visible light due to the charge transfer occurring at its surface. At the same time, this material exhibited a specific exposure of reactive facets, enhancing interactions with target dyes of a different nature. The results on adsorption and photodegradation performance of both nanomaterials demonstrate that the functionalization of morphologically evolved TiO_2_ NPs is the key strategy to expand applicability in advanced photocatalytic and energy-related processes, while also tailoring the surface for specific chemical interactions with organic pigments.

## Experimental Section

### Reagents and Solvents

The solvents utilized in this study were obtained from commercial suppliers and used without further purification. The synthesis and functionalization of oleic-acid-capped TiO_2_ nanorods were carried out using the following reagents: oleic acid (OA, Sigma-Aldrich, 90%), titanium­(IV) isopropoxide (TTIP, Sigma-Aldrich, 97%), trimethylamine N-oxide dihydrate (TMAO, Sigma-Aldrich, 98%), and 1,2-dihydroxybenzene (Cat, Sigma-Aldrich, ≥99%). Aeroxide P25 (TiO_2_, Thermo Scientific, 99.5%) was employed as a reference benchmark for the TiO_2_ nanomaterials. Removal experiments were conducted in deionized water (Milli-Q grade) using the following dyes: Methylene Blue (MB, Sigma-Aldrich, ≥97%), Rhodamine B (RhB, Sigma-Aldrich, fluorescence grade), Eosin B (EoB, Sigma-Aldrich, 90%), Rose Bengal (RB, Sigma-Aldrich, 95%), and Methyl Orange (MO, Sigma-Aldrich, 85%).

### Synthesis of OA-Capped TiO_2_ Nanorods (NR-OA)

Under an inert dinitrogen atmosphere, 25 mg of OA is kept under stirring at 120 °C (reflux) in a three-neck flask for 1h. The temperature is then reduced to 100 °C and 6.5 mmol of TTIP is added, causing a gradual change in color from colorless to pale yellow. After 5 min of stirring, 7.5 mL of a 2 M TMAO aqueous solution is rapidly added to the mixture starting the hydrolysis. The solution was stirred at 100 °C (reflux) for 25 h. The TiO_2_ NPs are precipitated with ethanol and subsequently purified through two washing cycles. Each cycle involves resuspension in toluene, precipitation with ethanol, and centrifugation. After drying *in vacuo*, the material was obtained as a lightly colored amber/yellow powder and stored in air.

### Functionalization of NR-OA with Cat (NR-Cat)

A suspension of 200 mg of NR-OA in 0.8 mL of chloroform is prepared in a centrifuge tube, to which 0.1 mL of a 10 M Cat solution in acetone is added. Upon addition, the suspension undergoes an immediate color change from pale yellow to red-brown. The mixture is then sonicated for 1 h at 40 °C. The NPs are precipitated using an excess of acetone and purified through four washing cycles, each consisting of resuspension in chloroform, precipitation in acetone, and centrifugation. The resulting rusty-red powder is dried and stored in air.

### Characterization

Thermogravimetric analysis (TGA) was carried out on a Q600 TA Instruments under a nitrogen flow (100 mL/min) at a temperature scan of 10 °C/min.

X-ray photoelectron spectroscopy (XPS) was carried out with a PHI 5000 Versa Probe II spectrometer (Physical Electronics) equipped with a monochromatic Al Kα X-ray source (1486.6 eV), operated at 15 kV and 24.8 W, with a spot size of 200 μm. Survey (0–1200 eV) and high-resolution spectra (C 1s, O 1s, Ti 2p) were recorded in Fixed Analyzer Transmission mode at a pass energy of 117.40 and 29.35 eV, respectively. Surface charging was compensated by means of a dual beam charge neutralization system, with a combination of a flux of low energy electrons (∼1 eV) and a one of low energy Ar^+^ ions (10 eV). The correction for the binding energies charging was carried out with respect to the C 1s core level line at 285.0 eV. All spectra were collected at an angle of 45° with respect to the sample surface. Curve fitting of C 1s was carried out with MultiPak data processing software (Physical Electronics), using a Shirley background, a 80% Gaussian/Lorentzian peak shape, and a FWHM of 1.4 eV.

Transmission Electron Microscopy (TEM) analysis was performed using a JEOL JEM1011 microscope operating at an accelerating voltage of 100 kV, equipped with a tungsten electron source and a high-resolution CCD camera for image acquisition. Samples for TEM were prepared by depositing 3 μL of a diluted sample solution (0.25 mg/mL) in either chloroform or ethanol onto a carbon-coated copper grid and allowing it to dry. TEM images were quantitatively analyzed by using ImageJ software. The mean length, diameter, and corresponding standard deviations were calculated based on measurements from a sample of 100 individual particles.

UV–vis spectroscopy in solution and solid-state diffuse reflectance spectroscopy (DRS) were conducted using a V-660 Jasco UV–vis/NIR Spectrophotometer. For DRS measurements, the instrument was equipped with a 60 mm integrating sphere (Jasco ISN-923) and a powder sample holder (Jasco PSH-001). Data acquisition and analysis were carried out using Spectra Management software to elaborate the acquired reflectance in absorbance values. The indirect band gap energy (*E*_g_) of the semiconductor was determined using the Kubelka–Munk function, followed by analysis with a Tauc plot. In this approach, (*F*(*R*) × *hν*)^1/2^ was plotted against the photon energy (hν).

FT-IR measurements were recorded on a JASCO 4200 spectrophotometer in the attenuated total reflectance (ATR) mode.

ζ-potential measurements were performed using a Malvern Zetasizer Nano ZS on 0.1 mg/mL water suspensions of each nanomaterial (P25, NR-OA, and NR-Cat).

### Adsorption Tests A - Adsorption Capacity

To evaluate the adsorption capacity of nanomaterials P25, NR-OA, and NR-Cat, MB was selected as the benchmark molecule. A 3 mL suspension of nanomaterials (1 mg/mL in water) was prepared, and MB was added to achieve a final concentration of 0.06 mM. The resulting suspension was stirred overnight (ON) for 16 h in the dark. The powders and liquids were separated via centrifugation. The powders were then dried under vacuum and analyzed by DRS, before and after their direct exposure to visible light for 2 h. The liquid samples, denoted as P25_ON, NR-OA_ON, and NR-Cat_ON, were collected and analyzed by UV–vis spectroscopy. The absorbance at λ = 661 nm was monitored to follow the decrease in the concentration of MB in solution, thereby evaluating its adsorption onto the nanomaterials.

### Adsorption Tests B - Nanomaterial-Dye Interaction

A similar procedure was applied to MB, RhB, MO, RB, and EoB, with the absorbance measured at their characteristic wavelengths. For each dye, a 3 mL suspension (1 mg/mL) of NPs in water was prepared, and the dye was added to achieve a final concentration of 0.05 mM. The suspension was stirred in the dark, and at regular intervals (every 10 min up to 60 min), it was centrifuged. The supernatant was collected and analyzed using a Jasco V-660 UV–vis/NIR Spectrophotometer V-660. The data were processed with Spectra Management software to monitor the decrease in dye concentration and evaluate its adsorption onto the nanomaterials. Consequently, the suspensions of the nanomaterials in the dye solutions were exposed to visible light using a UV–vis 300W Xe lamp (Quantum Design LS300Xe) equipped with a long-wave pass filter to block wavelengths below λ = 420 nm. The dye degradation was monitored for 30 min.

### Photocatalytic Tests

In a typical experiment, MB was added to a test tube containing 3 mL of a 1 mg/mL NPs suspension in water to reach a final concentration of 0.06 mM. The resulting solution was stirred in the dark for 60 min (conditioning step), after which visible light was applied using a UV–vis 300W Xe lamp (Quantum Design LS300Xe) equipped with a long-wavelength pass filter to block wavelengths below λ = 420 nm. Every 10 min, up to a total of 60 min of light exposure, the suspension was centrifuged, and the liquid phase was collected and analyzed by UV–vis monitoring the absorbance at λ = 661 nm, as described above. The degradation percentage, evaluated from the degree of discoloration,^28^ was calculated using the following equation

$$de\mathrm{grad}ation\;\%\;of\;MB=[100-\left(\frac{{{Abs}_{t}}^{\;}\times 100}{{Abs}_{t0}}\right)]$$

where Abs_*t*0_ represents the absorbance measured after the conditioning step, and Abs*_t_* is the absorbance at a given time. The degradation kinetics were plotted as ln­(*C*/*C*_0_) versus time, and the rate constant (*k*) was determined by linear fitting assuming first-order kinetics. For experiments investigating the role of reactive oxygen species (ROS) in the photocatalytic mechanism, specific scavengers were introduced into the reaction mixture. Tert-butanol (0.02 mM) was used as a scavenger for ^•^OH radicals and *p*-benzoquinone (0.02 mM) as a scavenger for ^•^O_2_^–^ radicals. The experimental conditions remained the same as described above, with the NPs, MB solution, and scavenger all present in a total volume of 3 mL water. The suspensions were stirred in the dark for 60 min for equilibration, followed by 60 min of light irradiation. The MB degradation percentage was calculated as described above, and the influence of each scavenger on the degradation process was assessed by comparing the results to control experiments conducted in the absence of scavengers.

## Results and Discussion

The synthesis of the NR-OA material was performed by adapting a low-temperature procedure that employs OA as both the capping agent and the reaction solvent to obtain TiO_2_ NRs in the anatase phase.^29^ As shown in Figure 1a, NR-OA was obtained from a fast hydrolysis of titanium isopropoxide (TTIP) in the presence of OA, acting as a stabilizing ligand that selectively binds the TiO_2_ surface at specific crystallographic planes inducing growth anisotropy. The use of trimethylamine oxide (TMAO) promotes the transformation under mild conditions (100 °C). The material was obtained as an air-stable, amber-colored powder that can easily be dispersed in organic solvents (such as chloroform and toluene) due to the presence of long alkyl chains on its surface.

**Figure 1:**
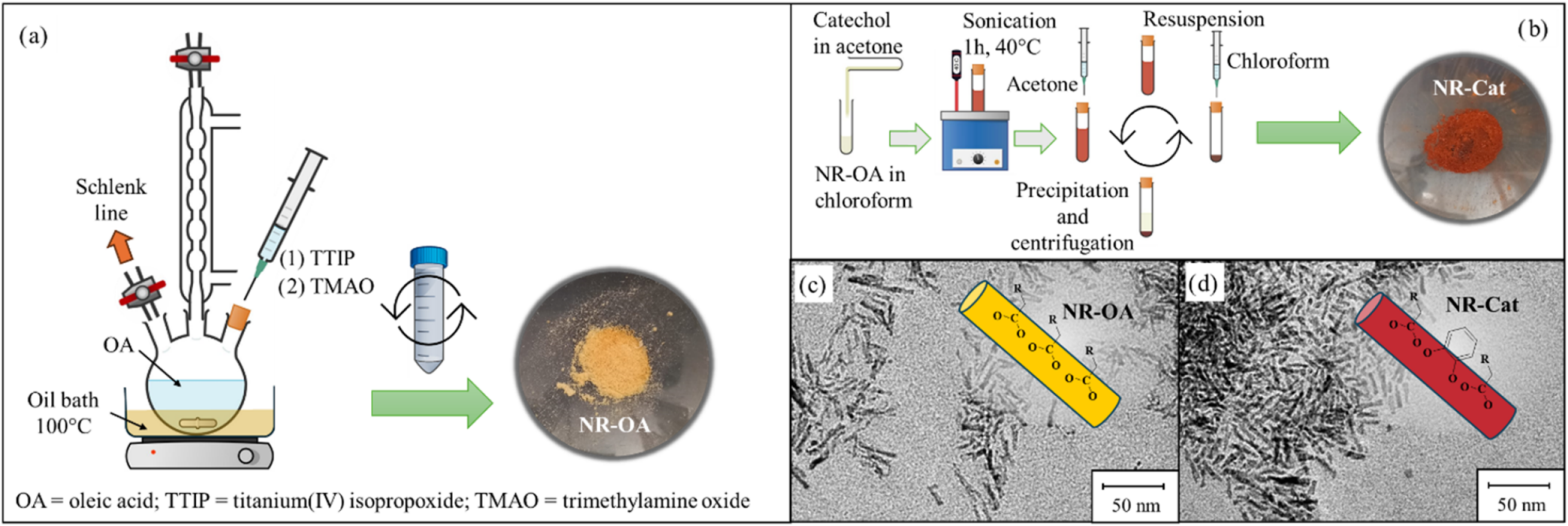
Schematic representation of the NR-OA synthesis (a) and the NR-Cat functionalization procedures (b). TEM images of NR-OA (c) and NR-Cat (d).

The functionalization with Cat was adapted from a procedure reported in literature^24^ and it is illustrated in Figure 1b. The procedure involves, as a first step, suspending hydrophobic NR-OA in a low-polarity solvent such as chloroform, thereby allowing an efficient dispersion. The required amount of Cat (as detailed in the Experimental Section) was dissolved in the proper amount of acetone, and the solution was added to the NR-OA suspension. Upon addition, a noticeable color change immediately occurred, shifting from amber to red-rust, indicating the formation of a charge-transfer complex following the ligand adsorption.^30^ Among the wide range of sensitizers, Cat is a small and easy to handle molecule with low toxicity, that can strongly bind the TiO_2_ surface through nature-inspired mechanism.^31^ With its two hydroxyl groups, Cat can be adsorbed on the TiO_2_ surface following a dissociative mode and therefore covalently bound as chelate, monodentate, or bidentate.^32,33^ The dissociative mode can introduce a high number of states in the semiconductor band gap, resulting in effective electron transfer processes, especially for the bidentate configuration that allows a stronger chromophore-semiconductor connection.^34,35^ Cat can consequently act as sensitizer allowing both type I and type II charge transfer and inducing an absorbance red-shift of the inorganic semiconductor.^23,36^

Efficient ligand exchange was ensured through sonication of the mixture, followed by washing steps to remove unbound OA and excess Cat. Solvent removal allowed the obtainment of a powder (NR-Cat) exhibiting an intense rust-like color, distinctly different from that of NR-OA. With reference to the catechol-modified NRs, these showed a slight improvement in water dispersibility that might be explained by invoking partial substitution of the long hydrophobic alkyl chains of the OA molecules, with the more polar Cat, increasing the material affinity for water. The incorporation of Cat at the nanoparticle surface was confirmed by FT-IR analysis (Figure S1), which revealed a broad absorption band attributable to the stretching vibrations of −OH groups.

TEM was employed to confirm the morphology of the material on the nanoscale. As evident from the inspection of Figure 1c, TEM micrographs revealed that the NR-OA sample has a nanorod morphology with an average length of 23.0 ±  3.0 nm and a width of 4.1  ±  0.5 nm, resulting in an aspect ratio of 5  ±  2. TEM analyses of NR-Cat confirmed that the nanorod shape of the material remains unchanged subsequently with the functionalization procedure. The NR-Cat showed an average rod length of 28.0 ± 3.0 nm and width of 5.3 ± 0.7 nm, confirming the 5  ±  2 aspect ratio (Figure 1d).

To gain insights into the surface modification, TGA was carried out on both the NR-OA and NR-Cat samples (Figure 2a). The TGA profile of NR-OA showed a single thermal event corresponding to the weight loss of the only organic component on the nanomaterial (OA). The onset temperature of this degradation is evaluated to be 248 °C, and the process was completed at 450 °C. Conversely, the thermogram of NR-Cat displayed two distinct thermal events. The first one, with an onset temperature at 198 °C, can be correlated to the weight loss of Cat, while the second one, starting at 329 °C, can be associated with the loss of OA. In fact, a control experiment was performed by subjecting pure OA and Cat under the same conditions of the NPs, revealing thermal events leading to weight loss (incipient evaporation) at 160 °C and 90 °C, respectively. The absence of corresponding events at these temperature values in the thermograms of NR-OA and NR-Cat suggests that negligible amounts of unbound OA and Cat are present on the nanomaterial surfaces, while the presence of two thermal events suggests the coexistence of both bound ligands (OA and Cat) on the NR-Cat surface. Indeed, Cat has also been demonstrated to partially displace OA from the surface of hydrophobic TiO_2_ nanospheres.^37^ Moreover, the residual weight percentage at 500 °C (corresponding to relative weight of the inorganic core) is higher for NR-Cat than for NR-OA (vide infra), supporting the partial substitution of OA with catechol on the nanocrystal surface. This is explained by the molecular weights of the ligands: OA is, in fact, significantly heavier (282.46 g/mol) than catechol (110.1 g/mol). Consequently, the replacement of some molecules of oleic acid with the lighter catechol reduces the overall weight loss during thermal decomposition, resulting in a higher residual inorganic weight for NR-Cat.

**Figure 2:**
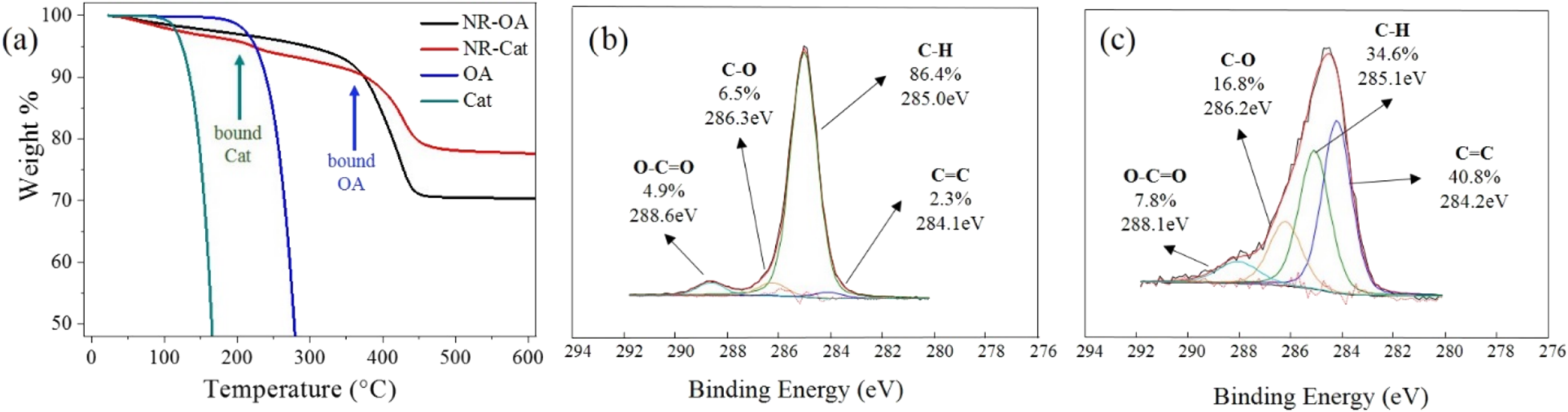
(a) Thermograms for NR-OA, NR-Cat, as well as OA and Cat; (b) XPS C 1s signal for NR-OA; (c) XPS C 1s signal for NR-Cat; XPS signal best fitting emphasizes the decrease of C–H component due to substitution.

The weight losses observed in TGA allowed quantification of each organic species on the NR, reported in Table 1 as μmol of substance per mg of TiO_2_. In addition, these data have been correlated with the NRs dimensions calculated by TEM and, considering the reported density for the anatase phase (*d* = 3.9 g/cm^3^), the ligand density for OA and catechol was calculated and expressed as molecules of ligand per nanorod surface area (Table 1). These results indicate that 32.4% of the OA molecules on the surface were replaced by Cat, leading to a slight increase in the total ligand density from 3.30 to 3.58 molecules/nm^2^. This result is in accordance with the partial substitution reported by Schechtel et al., who observed that only 20% of the OA originally forming the shell surrounding the TiO_2_ nanospheres was displaced by Cat.^37^

**Table 1:** Estimated Amounts of OA and Cat on the NRs Structure, Expressed as μmol/mg of TiO_2_, along with Ligand Density Values (molecules/nm^2^) for NR-OA and NR-Cat

samples	NR-OA	NR-Cat
OA (μmol/mg TiO_2_)	1.43	0.75
Cat (μmol/mg TiO_2_)		0.45
ligand density OA (molecules/nm^2^)	3.30	2.23
ligand density Cat (molecules/nm^2^)		1.35

Given the critical importance of surface characteristics in determining the properties and behavior of nanorods, an XPS analysis of the nanorod materials (NR-OA and NR-Cat) surface was carried out, aimed at identifying the nature of organic species onto the TiO_2_. In particular, the C 1s signal was observed and appropriately best fitted for both NR-OA and NR-Cat. The analysis reveals a few significant differences between the two samples (Figure 2b,c) and notably a marked decrease in the intensity of the C–H signal at a binding energy (BE) of 285 eV in NR-Cat, which is consistent with the partial substitution of OA, bearing a C-18 hydrocarbon chain, with the small aromatic ring of catechol. This observation aligns with the reduction in the C/Ti ratio from 4.1 to 2.6 observed in the atomic percentage analysis (Table S1), Conversely, an increase in the intensity of the C=C signal (284.2 eV) and the C–O signal (286.2 eV) can be observed, indicating the presence of catechol, characterized by its aromatic ring and C–O bonds. It is interesting how the BE of the C–O signal is slightly lower than expected for the C–OH of unbound catechol (286.5 eV), suggesting a possible interaction with titanium as an electron acceptor on the nanorod surface. Furthermore, analysis of the Ti 2p signals (Figure S2) confirm that the BE values for NR-OA and NR-Cat remain consistent with those reported for TiO_2_ in the anatase phase, supporting the preservation of the nanorod crystalline structure during the functionalization process. To exclude the possibility of nitrogen doping of the TiO_2_ lattice due to nitrogen incorporation from the catalyst (TMAO), we analyzed the N 1s XPS spectra of both NR-OA and NR-Cat. The results revealed that the atomic percentages associated with the N 1s peaks (0.6% for NR-OA and 1.1% for NR-Cat, Figure S3) are close to the detection limit. Furthermore, the N 1s peak appears at 401.5 eV in both samples, a binding energy characteristic of nitrogen in the ammonium state (likely originating from residual surface-bound reagents), thus ruling out nitrogen incorporation into the TiO_2_ structure as substitutional or interstitial doping agent.

The absorption spectra of the nanomaterials were measured by UV–vis DRS and are shown in Figure 3a. While it is well-known that P25 titania does not absorb in the visible range and appears as a white powder, the UV–vis analysis reveals that NR-OA exhibits a weak absorbance in the visible range, consistently with previous reports^38^ attributing this property to the ability of OA to act as a sensitizer for TiO_2_ NPs. Moreover, a markedly red-shifted and broad absorbance is recorded for NR-Cat, falling in the visible range. These observations are consistent with the amber/yellow color of the synthesized NR-OA material (shown in Figure 1a) and with the rust/red color of the NR-Cat powder (shown in Figure 1b).

**Figure 3:**
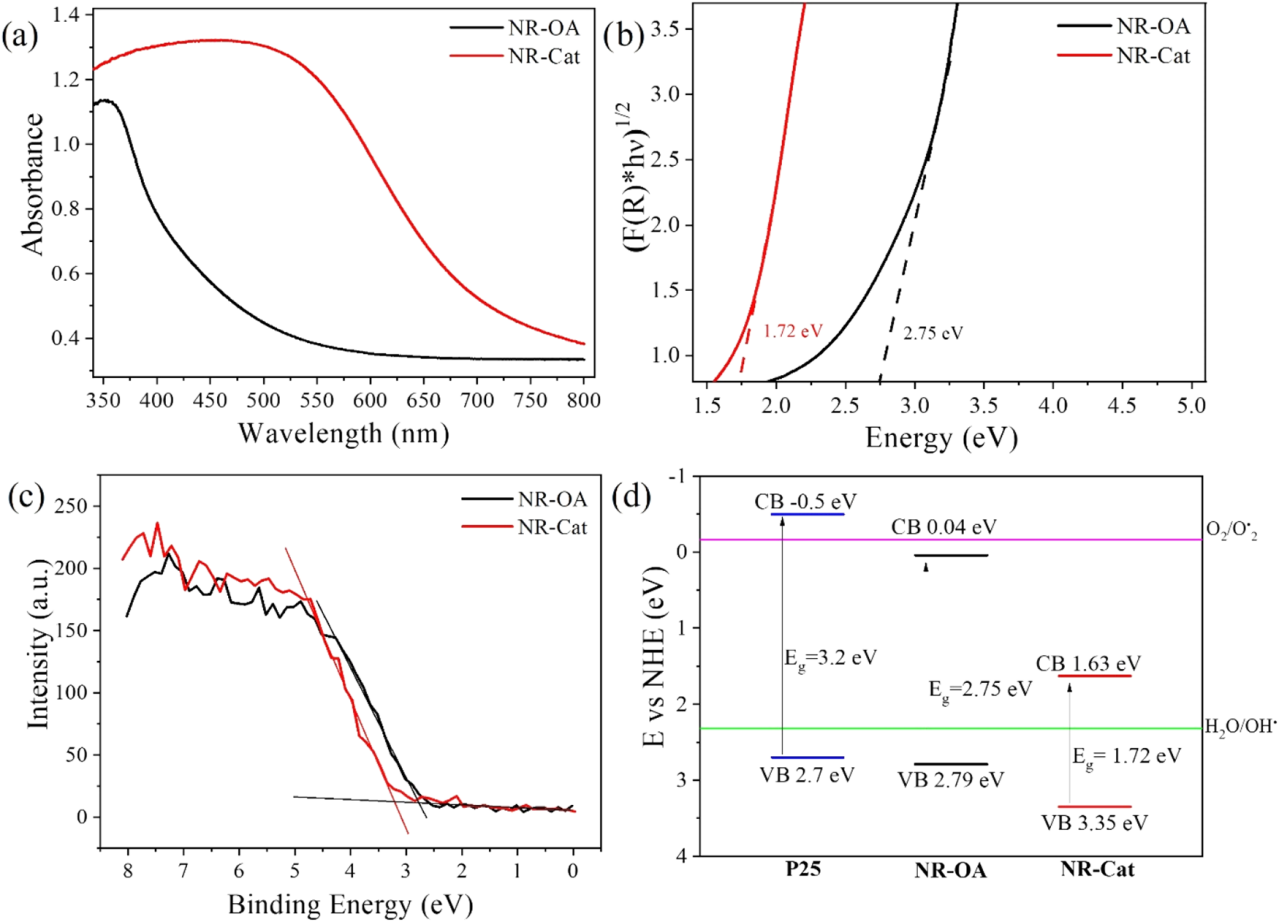
(a) DRS spectra of NR-OA and NR-Cat samples; (b) relevant Tauc plot with the corresponding indirect band gap values; (c) Plot showing the calculation of the Valence Band Maximum (VBM) for NR-OA and NR-Cat using the tangent method from XPS data; (d) energy level diagram reporting the valence band (VB), conduction band (CB), and band gap (*E*_g_) for NR-OA and NR-Cat, compared with analogous literature values for P25, as well as values for *E*H_2_O/^•^OH^0^ and *E*O_2_/^•^O_2–_^0^ potentials.

From the reflectance spectra of the nanomaterials, the Tauc plot was derived to determine the indirect band gap (Figure 3b). The band gap reported in the literature for P25 TiO_2_ is 3.2 eV and the same value was confirmed in this study (data not shown). For NR-OA, the band gap is reduced to 2.75 eV and it decreases further (1.72 eV) for NR-Cat. These values indicate that irradiation with visible light with wavelength above 451 nm for NR-OA and 721 nm for NR-Cat can generate the charge carriers.

Furthermore, XPS is a key technique for analyzing the electronic properties of materials, particularly the valence band maximum (VBM), which, along with the band gap (obtained by DRS), allows determination of the conduction band minimum (CBM), enabling a correlation between the electronic structure and activity, thereby providing a rationalization of the photoactivity. Therefore, the valence band maximum (VBM) for NR-OA and NR-Cat was determined using the XPS data shown in Figure 3c, while the conduction band minimum (CBM) was calculated by subtracting the corresponding band gap energy, obtained through DRS measurements, from the VBM (CBM = VBM – *E*_g_). The overall energy level diagram is shown in Figure 3d.

The energy levels determined for NR-OA and NR-Cat are depicted in Figure 3d, with the energy levels of P25 TiO_2_, as reported in the literature,^39^ included for comparison. Since the degradation of MB primarily relies on the generation of two reactive oxygen species (ROS), ^•^OH and ^•^O_2_^–^ radicals, the potentials of the associated half-reactions (see below) are also presented in Figure 3d.

H2O+h+⇄OH•+H+⁣(EH2O/OH•0=+2.32V)

O2+e−⇄O2−•⁣(EO2/O•2−0=−0.16V)

It can be observed that alongside the previously noted reduction in *E*_g_ values passing from P25 to NR-OA and further to NR-Cat, there is also an increase in both the VB and CB energy levels. Importantly, all three samples feature a VBM with sufficient potential to drive H_2_O oxidation, enabling the generation of ^•^OH radicals. Conversely, only P25 exhibits a CBM sufficiently low to facilitate the reduction of O_2_, leading to the formation of ^•^O_2_^–^ radicals. At the same time, a non-negligible fraction of the incident photons, those with energies close to 2.95 eV, corresponding to the cutoff wavelength of the optical filter used in the photocatalytic experiments (λ ≥ 420 nm), possesses sufficient energy to excite electrons from the valence band (VB) to conduction band (CB) levels capable of reducing molecular oxygen. Given that the VB edge of NR-OA is located at +2.79 eV vs NHE, excitation with photons of 2.95 eV results in photoexcited electrons reaching energy levels around −0.16 eV vs NHE, which coincides with the redox potential of the O_2_/^•^O_2_^–^ couple. This energetic alignment supports the feasibility of superoxide generation under the applied illumination conditions.^40^ With the same reasoning, the NR-Cat material shows a VB energy that would not allow the photogenerated electrons to reach the *E*_O2/^•^O2^–^_^0^ energy since it would require photons with λ = 354 nm or below. This band misalignment could imply that for NR-Cat only photogenerated holes (h^+^) are active and contribute to the photocatalytic degradation with ^•^OH formation but not the electrons, which would have contributed with ^•^O_2_^–^ production. This could, in turn, imply a lower photocatalytic activity for the NR-Cat sample compared to that of NR-OA, despite its higher adsorption capacity in the visible range (vide infra).

### Adsorption vs Photodegradation

To assess the adsorption capacity of the synthesized nanomaterials, relevant powdery samples of NR-OA and NR-Cat were suspended in a 0.06 mM aqueous solution of methylene blue (MB) and kept in the dark overnight (ON) under constant stirring. MB is the prototypical dye conventionally used to simulate water contamination with an organic pollutant in laboratory tests. At the end of the contact period, the suspensions were centrifuged to evaluate the absorbance of the supernatant liquids, monitoring the intensity of the MB characteristic absorption maximum at 661 nm. As shown in Figure 4a–c, all nanomaterials effectively reduced the MB concentration in the dark. The removal efficiency was found to be 23 ± 1%, 47 ± 2%, and 91 ± 4% for P25, NR-OA, and NR-Cat, respectively. A more direct comparison between the different nanomaterials was reported in Figure S4 for clarity. Based on the absorption measurements, the amount of MB adsorbed onto the nanomaterials was quantified (in terms of nanomoles of dye adsorbed per gram of powder) as 11, 33, and 58 μmol/g for P25, NR-OA, and NR-Cat, respectively. To exclude unexpected degradation processes alternative to adsorption, the UV–vis absorption spectra of the powders (denoted as P25_ON, NR-OA_ON, and NR-Cat_ON) separated by centrifugation at the end of the overnight adsorption were acquired by DRS. As displayed in Figure 4d–f, in addition to their own electronic transitions, all nanomaterials exhibited a further absorption band comprised between λ = 550 and 750 nm emerging after overnight contact with the MB solution. This broad band can be attributed to the MB absorption by comparison with its spectrum in aqueous solution and, combined with the color consistency of the recovered powders (insets of Figure 4d–f), indicates that the dye removal from the aqueous solution selectively occurs through the adsorption process on the semiconductor surface.

**Figure 4:**
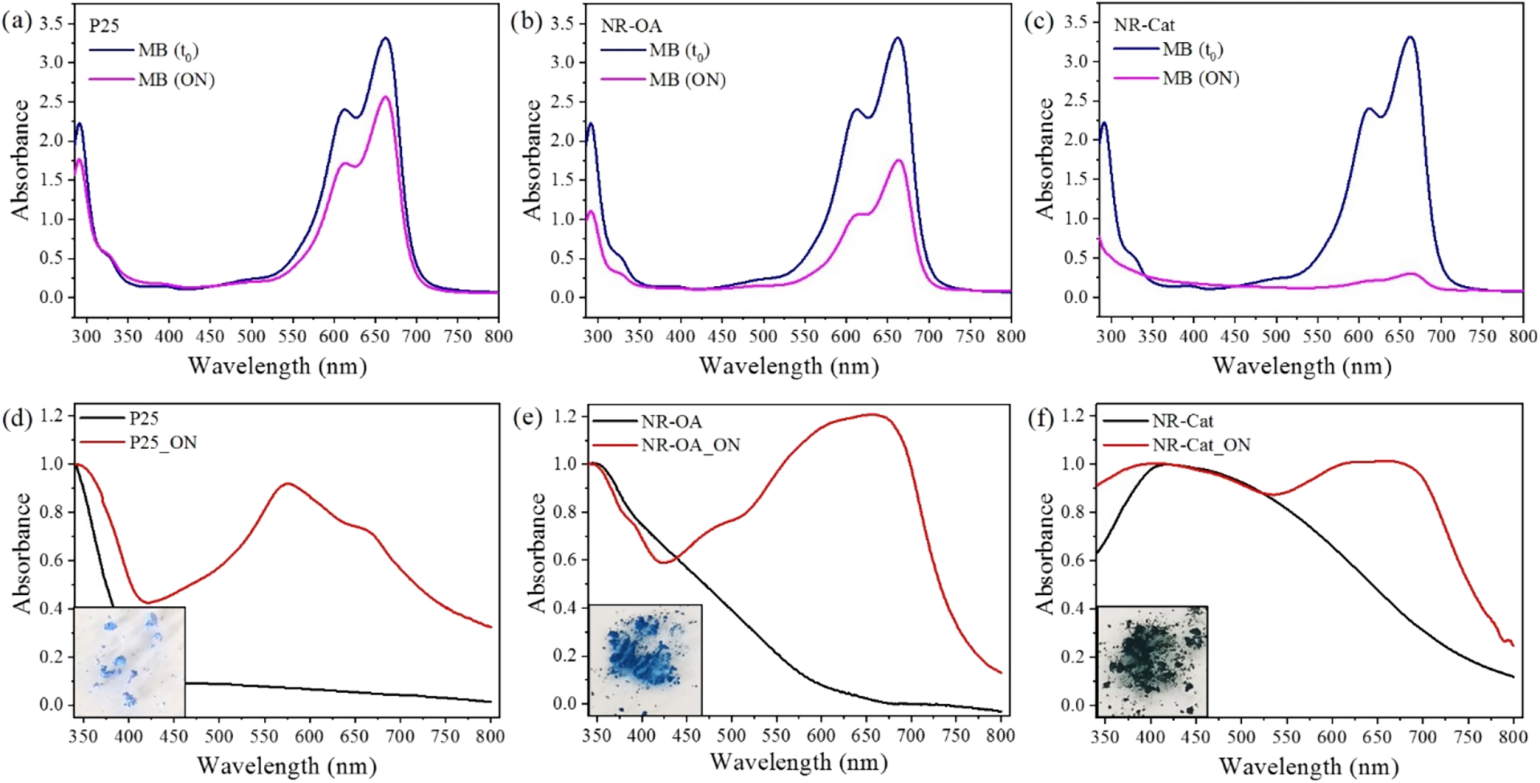
Absorption spectra of MB solution before (*t*_0_) and after overnight (ON) contact with (a) P25, (b) NR-OA, and (c) NR-Cat. DRS absorption spectra of the recovered powders after overnight (ON) contact with the MB solution for (d) P25, (e) NR-OA, and (f) NR-Cat in comparison to the absorption profiles of the pristine forms. The inset images in (d–f) show photographs of the corresponding powders (P25_ON, NR-OA_ON, and NR-Cat_ON).

A plausible explanation for this behavior could be related to the different electrostatic interactions between the nanomaterials surface and dyes scaffold. In fact, the ζ-potential of the samples at neutral pH was found to be negative for all three nanomaterials with its absolute value increasing in the following order: P25 ≪ NR-OA < NR-Cat (Table 2). The extremely low absolute value observed for P25 is consistent with its proximity to the isoelectric point in water.^4,41^ The negative charge of NR-OA and NR-Cat derives from residual Ti–OH groups on nanomaterials surface,^42,43^ which can be ascribed to the chemical approach (requiring hydrolysis of alkoxy derivative of titanium) used for their preparation. It can be noted that the surface modification slightly increased the surface negative charge of the relevant NPs, probably due to the insertion of monodentate catechol moieties.^44^

**Table 2:** ζ-Potential and Amount of MB Adsorbed per Gram of Powder for P25, NR-OA, and NR-Cat

samples	ζ-potential (mV)	MB adsorbed (μmol/g)
P25	–5.6 ± 0.4	11
NR-OA	–27.9 ± 0.5	33
NR-Cat	–31.0 ± 1.0	58

To support this interpretation, a systematic investigation on the nanomaterial adsorption propensity was carried out in the presence of differently structured dyes – namely RhB, EoB, MO, and RB – by attesting the evolution of their characteristic absorption profile in the relevant suspensions over a 60 min time span (shown in Figures S5 and S6). The selected dyes were chosen for their good solubility in water and to explore other parameters such as functional groups, scaffold structure, and charge at neutral pH. While RB and EoB belong to the xanthene-based family of pigments with a negative charge on the organic chromophore, RhB presents a positive charge on a similar xanthene structure. Conversely, MO is an anionic azo-benzene colorant. As evident in Figure 5, NR-OA and NR-Cat showed an adsorption capacity only for the cationic dyes (MB and RhB, Figure 5a,b), while for the anionic dyes (MO, EoB, and RB, Figure 5c–e), the nanomaterials did not show adsorption aptitudes.

**Figure 5:**
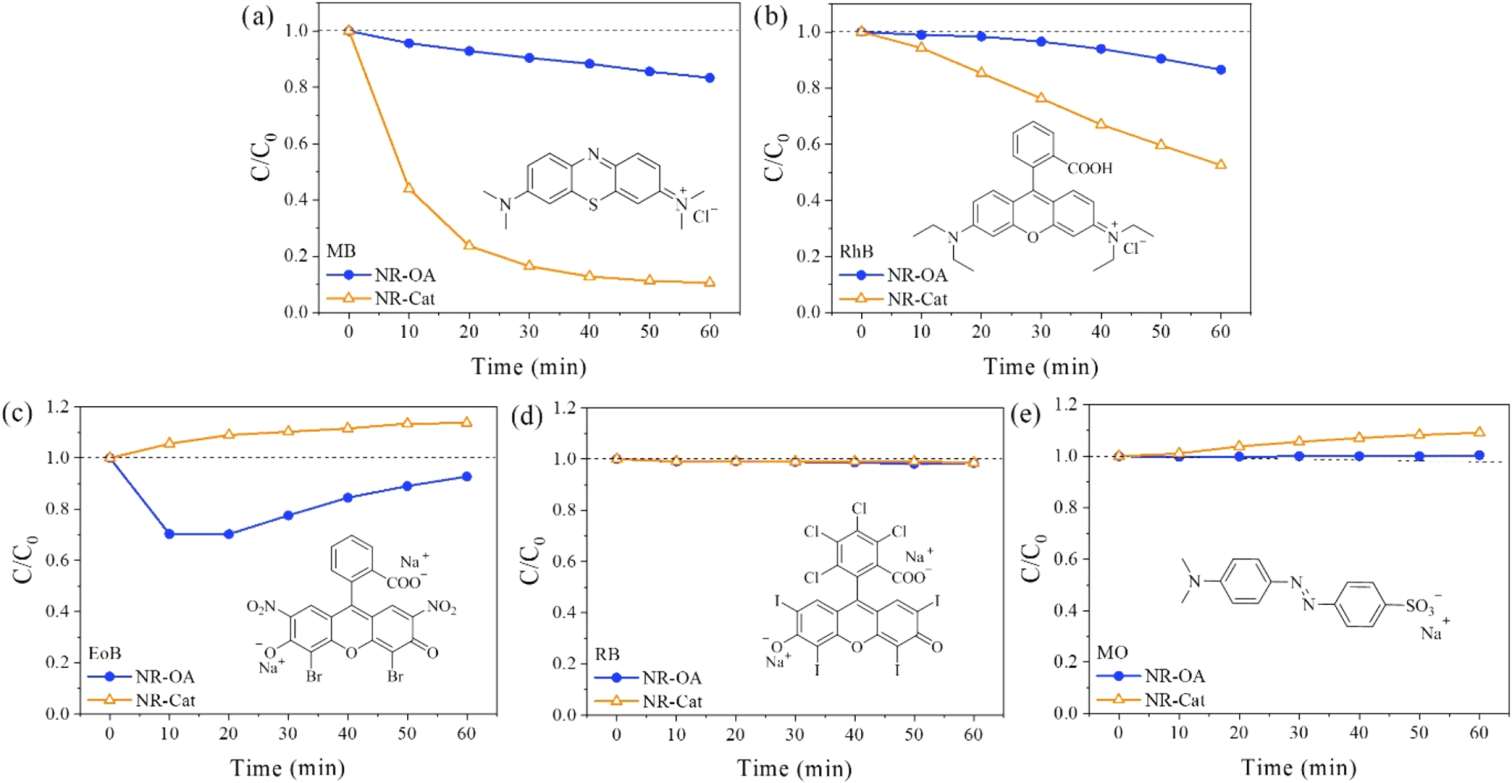
Adsorption profiles of NR-OA and NR-Cat for (a) MB, (b) RhB, (c) EoB, (d) RB, and (e) MO.

The entity of surface charge explains the enhanced adsorption of cationic dyes in NR-OA and NR-Cat, which can be attributed to attractive electrostatic interactions established between the (positive) colorants and (negative) nanomaterials. In fact, the adsorption of anionic pigments mediated by the three nanomaterials was negligible independently of the steric encumbrance of the dye structure or the chemical nature of the functional group characterizing the molecule. Moreover, the trend in the absolute value of the ζ-potential seems to be consolidated by the amount of adsorbed MB molecules (Table 2), but, at the same time, the organic shell compactness plays a crucial role in governing the entity of the dye adsorption. Being NR-Cat less hydrophobic than NR-OA, it can be in better contact with the dye molecules in solution and the π-π stacking interaction can further improve the adsorption of dyes containing aromatic rings.^45^

Comparing the adsorption of MB and RhB on NR-Cat a different behavior can be highlighted: even if the dyes are both cationic molecules, RhB is adsorbed in smaller amounts, probably due to a bigger steric hindrance correlated to the presence of an additional benzene ring (1.44 nm × 1.09 nm × 0.64 nm for RhB^46^ and 1.70 nm × 0.76 nm × 0.33 nm for MB^47^) that prevents the bulky and nonplanar molecule to intercalate and interact with catechol molecules on NR-Cat surface.

The slight enhancement of the absorbance value during the adsorption experiment carried out with NR-Cat in the presence of EoB or MO can be explained by the perturbation of the proton exchanges occurring at the NP surface during the adsorption/desorption equilibria. This behavior seems to be confirmed by the perceptible hypsochromic shift of the absorption maximum of MO solution (Figure S6). Nevertheless, the main outcome is that this azo-benzene anionic dye is not adsorbed by both NPs.

Although Brunauer–Emmett–Teller (BET) analyses could add relevant information to the synthesized materials, the results are not crucial to the rationalization of their adsorption (and photodegradation) behaviors. While differences in surface area between commercial P25 and the functionalized nanorods are expected, the specific surface areas of the synthesized NR-OA and NR-Cat samples are likely to be comparable, as they consist of similarly sized nanorods. Our interpretation of the dye adsorption results is based primarily on surface charge interactions rather than on total adsorption capacity, reasonably independent of the dye’s molecular structure. This is supported by the absence of adsorption for dyes bearing negatively charged organic moieties on both functionalized nanorods, indicating that electrostatic interactions dominate and that dye intercalation into the semiconductor lattice does not significantly contribute to the observed adsorption behavior. Moreover, the adsorption experiments were conducted under vigorous magnetic stirring, which likely disrupted the solid-state superstructure of the nanomaterials. Additionally, while the long organic chains on the nanorod surfaces may facilitate the diffusion of small molecules, such as nitrogen (used in BET analysis), they are unlikely to permit the penetration of larger organic dye molecules, which are expected to interact primarily with the external surface of the superstructured agglomerates. In fact, the apolar nature of the ligands should hamper water penetration into the internal sections of the materials.

A first evaluation of the NPs photoactivity was carried out by exposing the nanomaterials recovered after the adsorption test and that have been in contact with the MB solution overnight (P25_ON, NR-OA_ON, and NR-Cat_ON) to visible light for 2 h in air. From DRS spectra (Figure S7), it can be observed that the MB adsorbed is not degraded, therefore excluding the direct contribution of the charge carriers, h^+^ and e^–^, in the MB photodegradation. To further explore the nanomaterial-dye interactions, the photocatalytic degradation of MB in water was investigated, as reported in Figure 6a.

**Figure 6:**
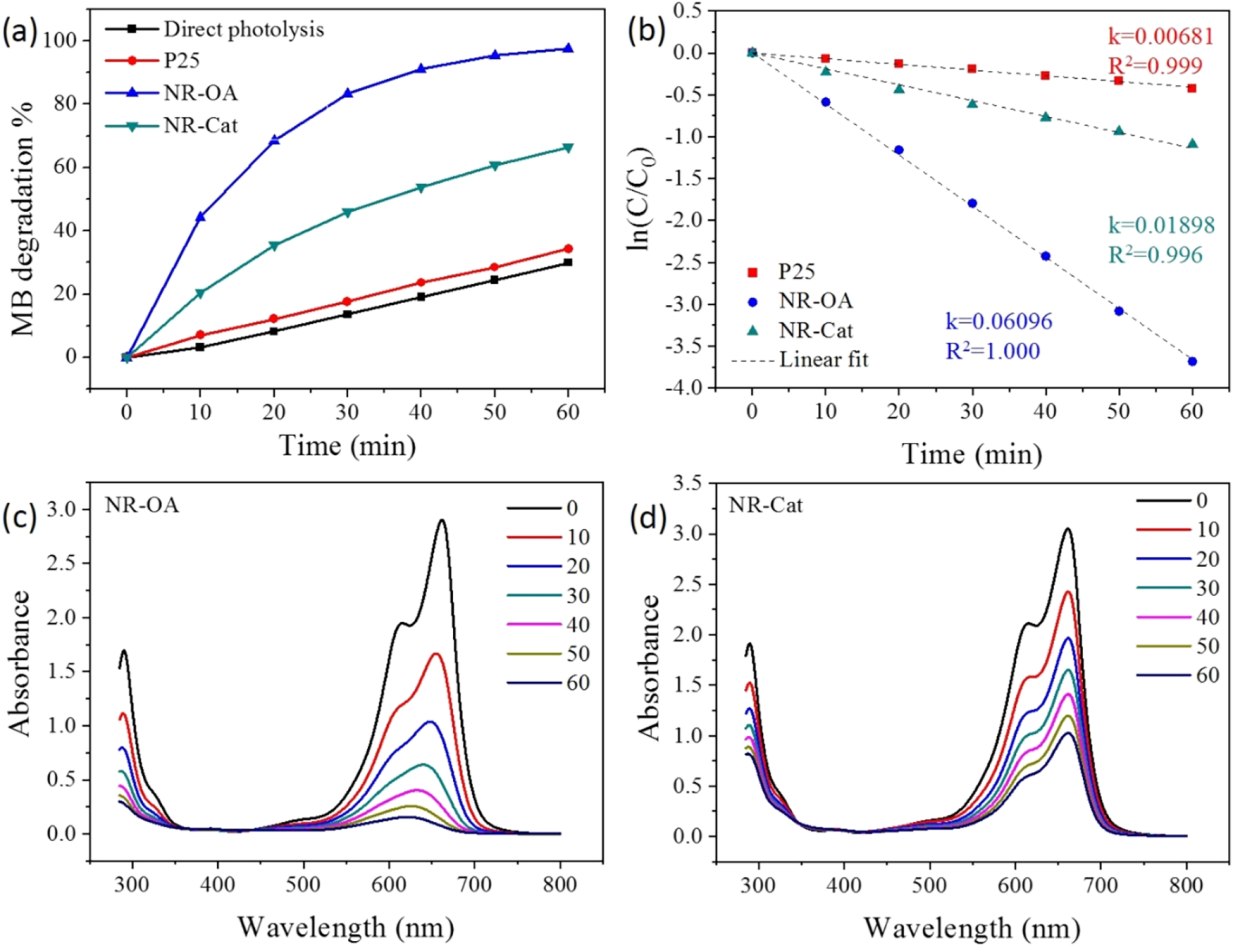
(a) Photocatalytic activity as degradation % of MB in water, (b) ln­(*C*/*C*_0_) vs *t* plot with pseudo-first order kinetic linear fit, (c) evolution of the MB’s absorption profile during photodegradation with NR-OA, and (d) evolution of the MB’s absorption profile during photodegradation with NR-Cat.

First, direct photolysis (in absence of the catalyst) resulted in a 30 ± 1% MB degradation after 60 min. Subsequently, P25, NR-OA, and NR-Cat were tested in the photocatalytic degradation of the same dye. These results were obtained after a conditioning step, during which the samples (see Experimental Section) were stirred in the dark for 60 min before irradiation with visible light. The use of P25 allowed the obtainment of a result, in terms of decomposition (34 ± 1%), close to the one recorded in the photolysis experiment. This very low photodegradation achieved by P25 is consistent with its negligible absorbance and limited photoactivity under visible light irradiation. In contrast, both nanorod samples showed an increased degradation ability. In particular, unfunctionalized NR-OA reached a plateau at 97 ± 5% photodegradation, proving its significantly enhanced activity under visible light. Conversely, NR-Cat achieved a 66 ± 3% degradation, a value lower than that of NR-OA although well above the performance of P25. The degradation profiles for NR-OA and NR-Cat were reported in Figure 6c,d, respectively.

The decomposition kinetics were analyzed transforming the concentration vs time plot into a ln­(*C*/*C*_0_) vs time, obtaining a linear fitting, according to a pseudo-first order decomposition kinetic (Figure 6b). The calculated kinetic constants are reported in Figure 6b and evidence a 3-fold value for NR-Cat (*k* = 0.01898 min^–1^) with respect to P25 (*k* = 0.00681 min^–1^), while the as-prepared nanorod exhibited the highest decomposition rate constant (*k* = 0.06096 min^–1^).

To further elucidate the photocatalytic mechanism and the roles of reactive oxygen species (ROS), scavenger tests were conducted to distinguish the contributions of ^•^OH and ^•^O_2_^–^ radicals in the degradation process, having previously excluded the direct involvement of h^+^ and e^–^. These experiments provide a deeper understanding of the active species involved and their correlation with the electronic structure of the nanomaterials, as discussed above. The scavenger tests were performed using *tert*-butanol as a hydroxyl radical scavenger and *p*-benzoquinone as a ^•^O_2_^–^ scavenger to selectively inhibit the activity of these ROS.

Figure 7 summarizes the results of the scavenger tests. It highlights how for NR-OA, the photocatalytic degradation of MB decreased from 97 ± 5%, as reported above, to 73 ± 4 and 59 ± 3% following the addition of scavengers for ^•^OH and ^•^O_2_^–^ radicals, respectively, demonstrating the involvement of both radicals in the degradation mechanism. Moreover, the results show that for NR-Cat, the MB degradation, from a value of 66 ± 3%, decreased to 46 ± 2% in the presence of the ^•^OH scavenger, while it remained almost unaltered in the presence of the ^•^O_2_^–^ scavenger (62 ± 3%), emphasizing that the degradation mechanism involves only the ^•^OH radical. This observation aligns with the considerations of the VB and CB energy levels presented in Figure 3d, which thermodynamically support the formation of both radical species for NR-OA and exclusively ^•^OH for NR-Cat.

**Figure 7:**
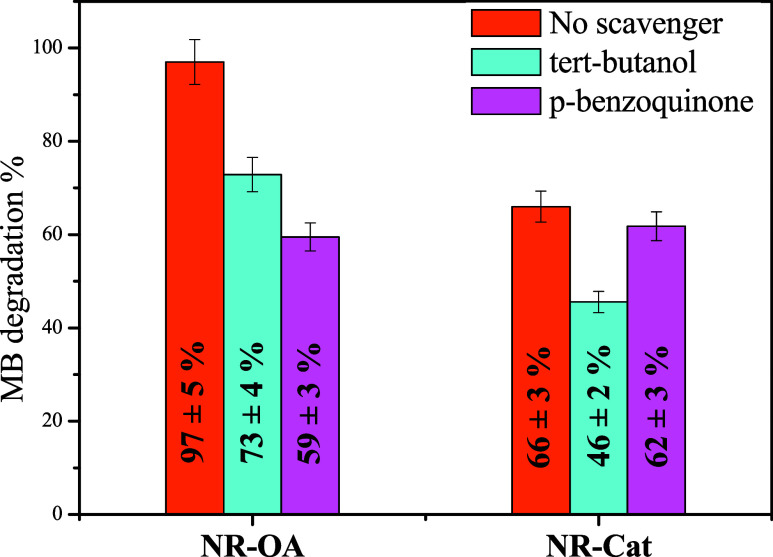
MB degradation percentages after 60 min of light irradiation for NR-OA and NR-Cat, obtained in the absence of scavengers, and in the presence of *tert*-butanol as an ^•^OH scavenger and p-benzoquinone as an ^•^O_2_^–^ scavenger.

The degradation of the other dyes was monitored for 30 min under visible light for NR-OA (Figure 8) and NR-Cat (Figure S8) after 60 min of adsorption/desorption equilibration in the dark. It can be observed that NR-OA efficiently decomposed EoB and RB with *k* = 0.11081 min^–1^ and *k* = 0.04975 min^–1^, respectively. In the case of RhB, we verified the hypsochromic shift on the absorption maximum (from 553 to 503 nm) that suggested the de-ethylation of the molecule,^50,51^ hampering the evaluation of the relevant kinetic constant. In addition, MO was not degraded by NR-OA. On the other hand, NR-Cat is not able to degrade MO and EoB, while exhibiting degradation activity for RhB (*k* = 0.01792 min^–1^) and RB (*k* = 0.00290 min^–1^).

**Figure 8:**
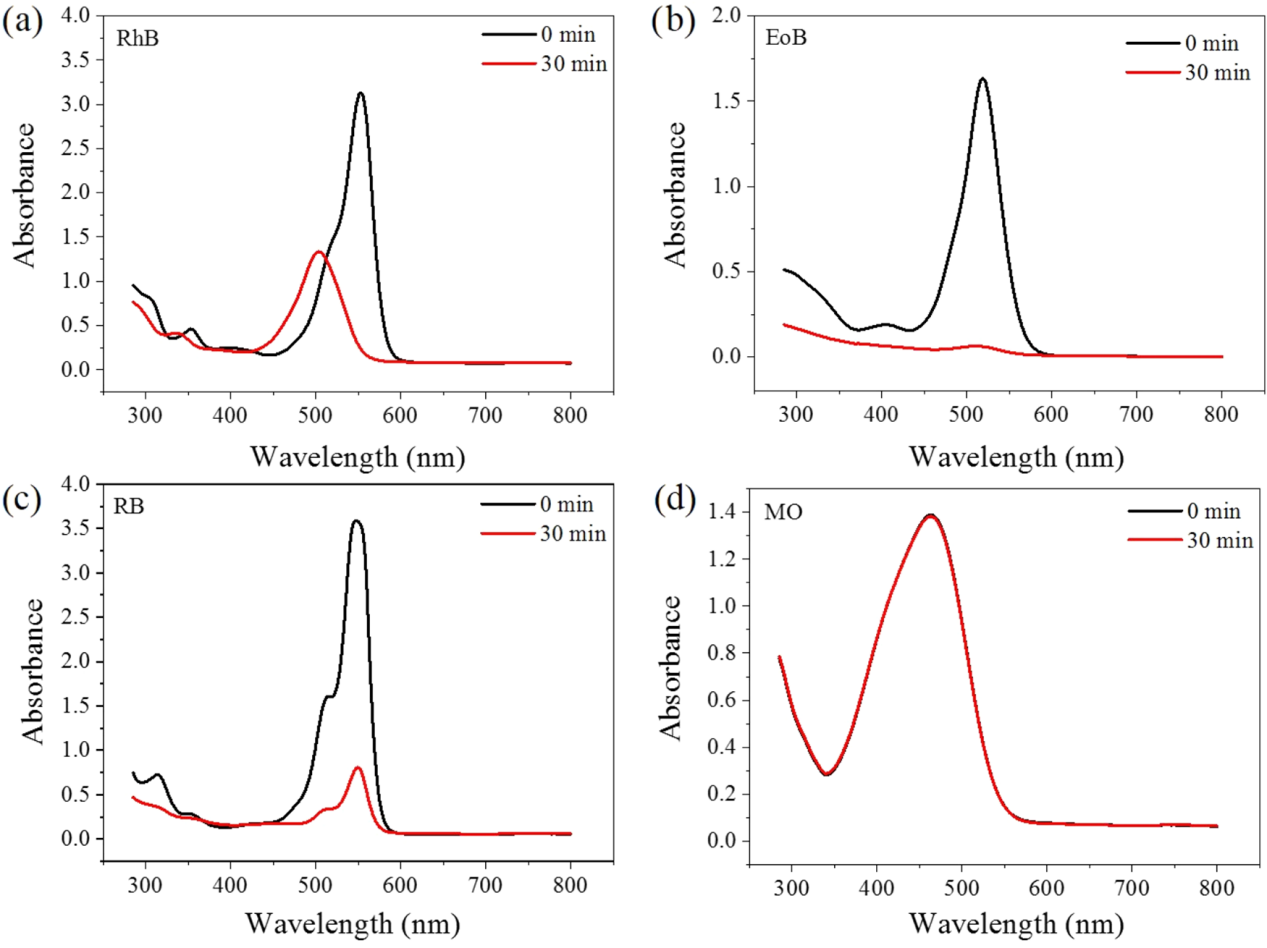
Absorption spectra before and after 30 min exposure to light in the presence of NR-OA of different dye solutions: (a) RhB, (b) EoB, (c) RB, and (d) MO.

Although electron spin resonance (ESR) and trapping experiments can help further identify the radical species involved in dye decomposition, we can already outline a possible photoinduced degradation pathway based on the information gained. We can state that

H2O+h+(TiO2VB)⇄OH•+H+

O2+e−(TiO2CB)⇄O2−•

O2−•/OH•+dye⇄degradationproducts

where the contribution of each ROS depends on the peculiar band alignment of the employed photocatalyst and decomposition rates are related to dye scaffold.

## Conclusions

Titanium dioxide NRs capped with organic ligands are widely recognized as versatile materials platforms for environmental applications. In this study, we advanced this approach by introducing a partial substitution of OA with catechol at the NR surface (NR-Cat), aiming at exploiting the strong catechol-TiO_2_ interactions to expand the NR functional properties. The partial substitution of OA (confirmed by TGA and XPS measurements) with catechol was evidenced by a distinct color change to red/rust, indicative of efficient charge transfer with a strong impact on the CBM position of the relevant nanomaterial. The surface chemistry modification strongly influences the efficiency and pathways of dye removal. For NR-OA, the most efficient strategy for dye removal was revealed to be photodegradation, although the adsorption process was still a viable option for MB removal (33 μmol/g compared to 11 μmol/g measured for P25). Among the other dyes tested, only a cationic dye (RhB) was absorbed, whereas the anionic dyes tested (EoB, RB, and MO) were not. This behavior is due to electrostatic interactions related to the negative surface charge (−27.9 ± 0.5 mV) of NR-OA measured with the ζ-potential. On the other hand, photodegradation is the predominant pathway for NR-OA, achieving nearly complete MB degradation after 60 min under visible light irradiation. The photoactivity under visible light is compatible with its reduced band gap energy of 2.75 eV (vs 3.2 eV for P25). The structure of the dye plays an important role in photodegradation since other xanthene dyes were degraded as well (RhB, EoB, and RB) while MO, an azo-dye, was not. For NR-Cat, the most efficient strategy for dye removal is adsorption rather than photodegradation. The MB overnight adsorption was considerably higher than for NR-OA (58 μmol/g) due to a higher negative charge measured (−31.0 ± 1.0 mV). Similarly, RhB was the only other tested dye adsorbed due to its cationic nature, although with lower efficiency than MB, suggesting an influence of the molecular structure and hindrance. NR-Cat, despite its efficiently reduced band gap (1.72 eV) and increased absorbance in the visible range, showed an MB photodegradation efficiency of 66% after 60 min that, although less efficient than NR-OA, remains significant when compared to the reference P25 (34%.) The degradation efficiency lower than that of NR-OA is also confirmed for the other dyes tested. This behavior was hypothesized to result from a combination of (i) higher steric hindrance caused by the higher absorption of MB molecules and (ii) different energy levels of CB and VB, which suggests that the photodegradation promoted by NR-Cat occurs only via ^•^OH radical, as hinted by the scavenging experiments. This study has therefore provided new insights into the surface chemistry consequent to the ligand exchange process at the surface of TiO_2_ NPs, demonstrating how surface functionalization dictates the balance between the adsorption and photodegradation processes. Catechol-functionalization enhances adsorption capacity, highlighting the potential of TiO_2_ NPs in removing cationic dyes from wastewater.

## Supplementary Material



## Notes

Additional experimental details and graphs including photographs of materials. (PDF)
