# Supplementary material for: Surface Modification of TiO2 Nanorods for Dye Removal: Photodegradation vs Adsorption Activity
Source: ACS Omega. 2025 Nov 3;10(44):52296–307. doi: 10.1021/acsomega.5c02952 (PMC12613128; doi:10.1021/acsomega.5c02952)
Supplement: Supplementary file 1 [file ao5c02952_si_001.pdf]

Surface Modification of TiO_2 Nanorods for Dye Removal: Photodegradation *vs* Adsorption Activity

Chiara Lo Porto,[a] *Daniele Conelli,*[a] *Carlo Nazareno Dibenedetto,*[b] *Marinella Striccoli,*[b] *Fabio Palumbo,*[c] *Roberto Grisorio**[,a] *and Gian Paolo Suranna*[a,d]

[a]Dipartimento di Ingegneria Civile, Ambientale, del Territorio, Edile e di Chimica (DICATECh), Politecnico di Bari, Via Orabona 4, 70125 Bari, Italy. E-mail: roberto.grisorio@poliba.it

[b]CNR IPCF – Istituto per i Processi Chimico Fisici, UOS Bari, Via Orabona 4, 70126 Bari, Italy.

[c]CNR-NANOTEC – Institute of Nanotechnology, UOS Bari, Via Orabona 4, 70126 Bari, Italy

[d]CNR-NANOTEC – Institute of Nanotechnology, c/o Campus Ecoteckne, Via Monteroni, 73100 Lecce, Italy.

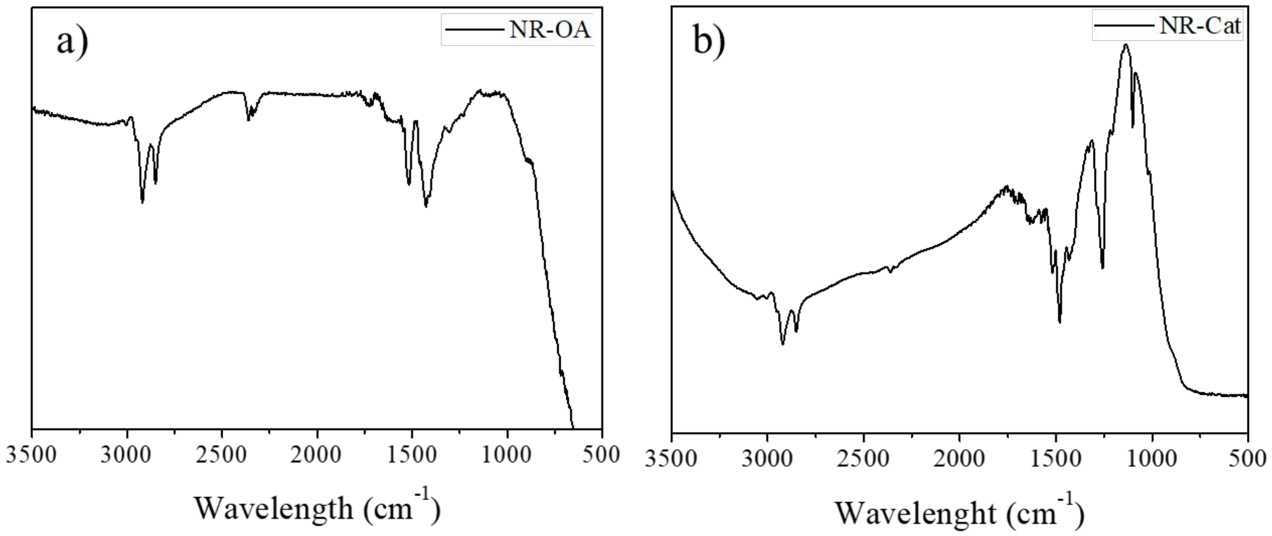

Figure S1. FT-IR spectra of NR-OA (a) and the relevant catechol-functionalized NR-Cat material (b).

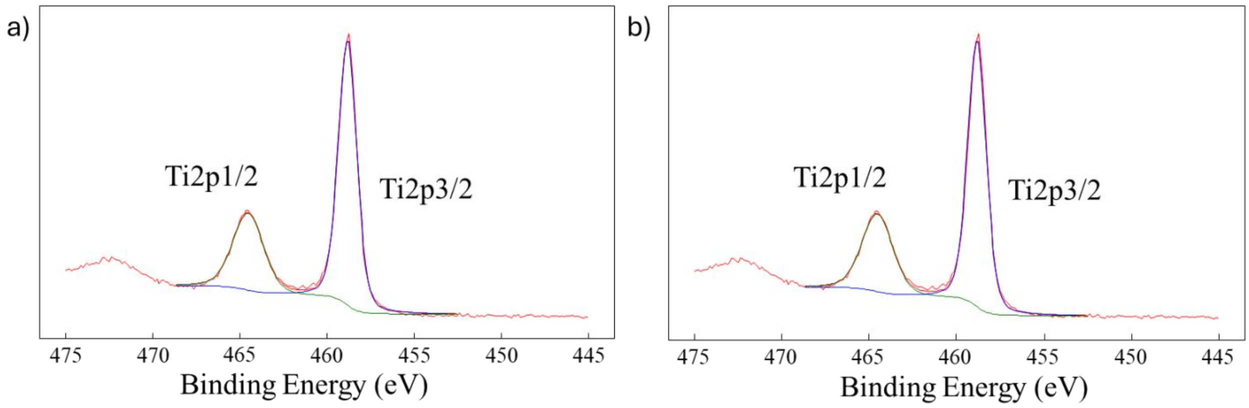

Figure S2. XPS Ti2p signals and their deconvolution for (a) NR-OA and (b) NR-Cat samples. For NR-OA, the Ti2p signal exhibits a Binding Energy (BE) of 458.8 eV for Ti2p$_{3/2}$ and 464.5 eV for Ti2p$_{1/2}$, with a spin-orbit splitting of 5.7 eV. For NR-Cat, the Ti2p signal shows a BE of 459.2 eV for Ti2p$_{3/2}$ and 464.9 eV forTi2p$_{3/2}$, also with a 5.7 eV splitting. These values are consistent with those reported for Ti(IV) in anatase TiO_2.

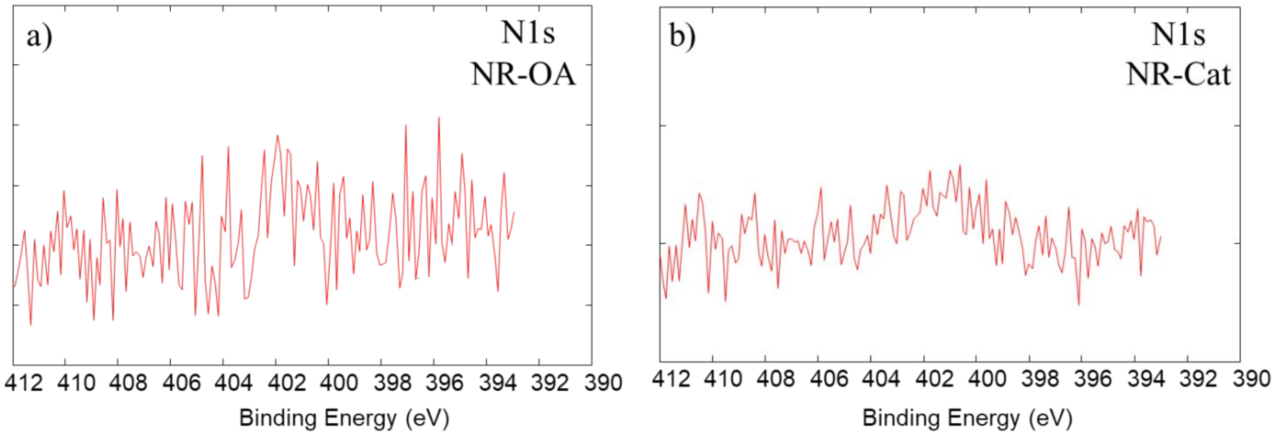

Figure S3. XPS N 1s signals of (a) NR-OA and (b) NR-Cat.

Table S1. Atomic percentage concentrations of Ti, O, and C for NR-OA and NR-Cat, obtained by XPS analysis. Additionally, the C/Ti and O/Ti ratios are reported for both samples. The partial substitution of the oleic acid carbon chain (18 carbon atoms) with the catechol aromatic ring (6 carbon atoms) in NR-Cat resulted in a reduction of the organic component, as shown by the decreased C/Ti ratio compared to NR-OA.

	**Ti %**	**O %**	**C %**	**C/Ti**	**O/Ti**
**NR-OA**	12.9 ± 0.6	34.5 ± 2.0	52.6 ± 3.0	4.1 ± 0.4	2.7 ± 0.3
**NR-Cat**	15. 9 ± 0.7	42.8 ± 2.0	41.2 ± 2.0	2.6 ± 0.2	2.7 ± 0.2

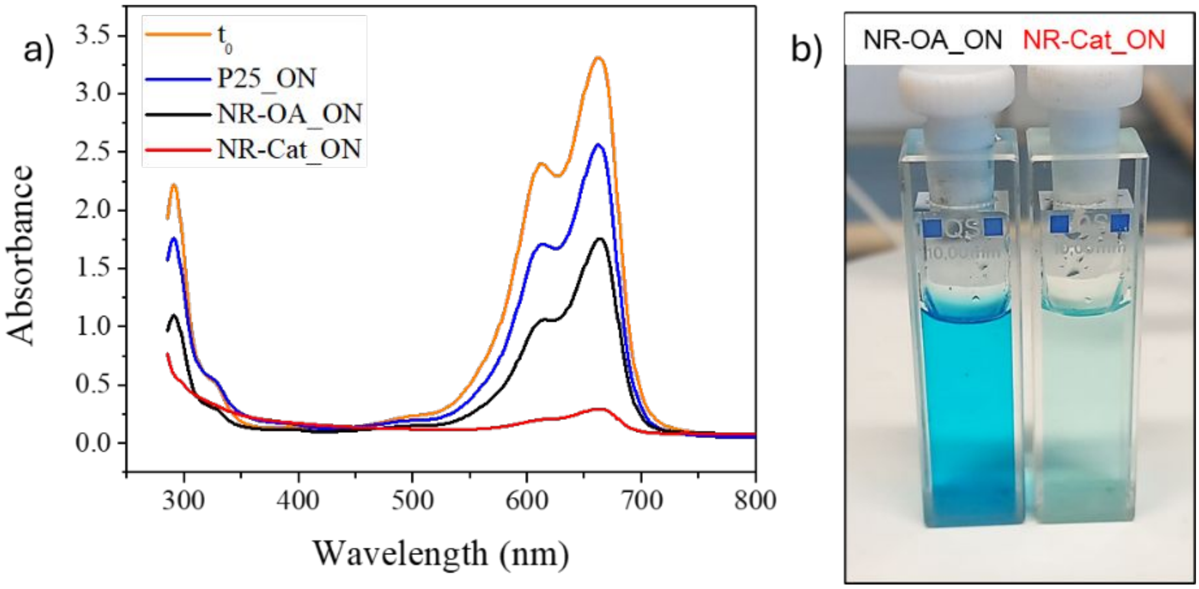

Figure S4. a) Absorbance spectra of methylene blue (MB) solution prior any contact with nanomaterials (t_0) and after overnight contact with P25, NR-OA and NR-Cat; b) photo of MB solutions after overnight contact with NR-OA and NR-Cat.

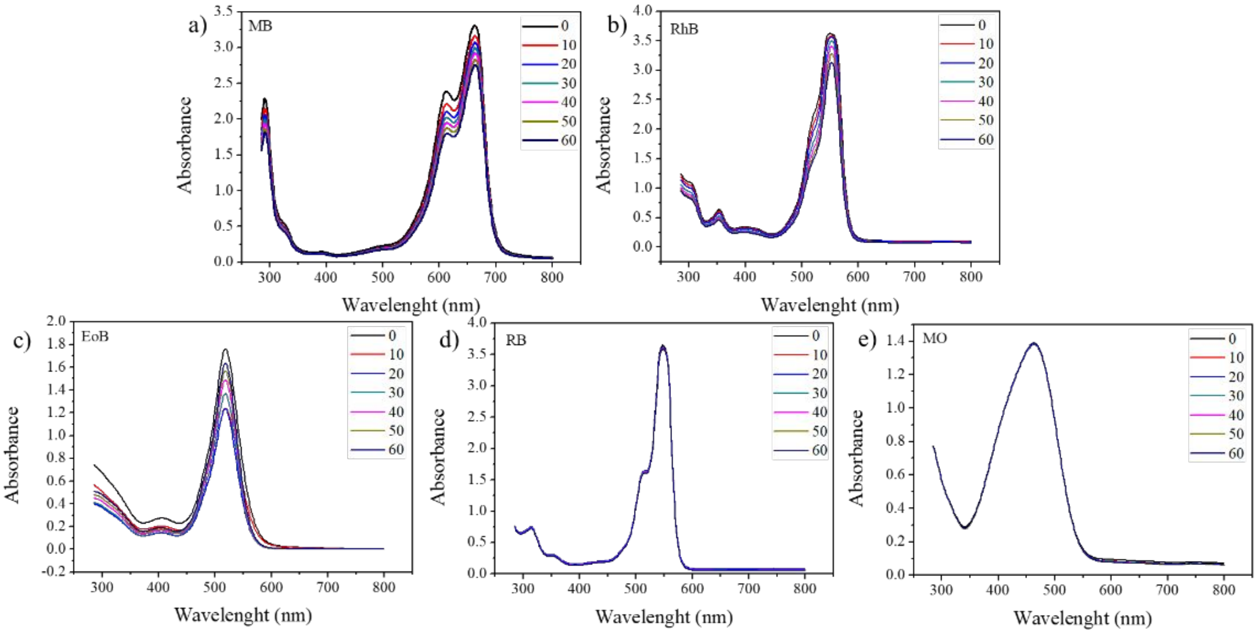

Figure S5. NR-OA adsorption profiles for different dyes: a) methylene blue (MB), b) rhodamine B (RhB), c) eosin B (EoB), d) rose Bengal (RB) and e) methyl orange (MO).

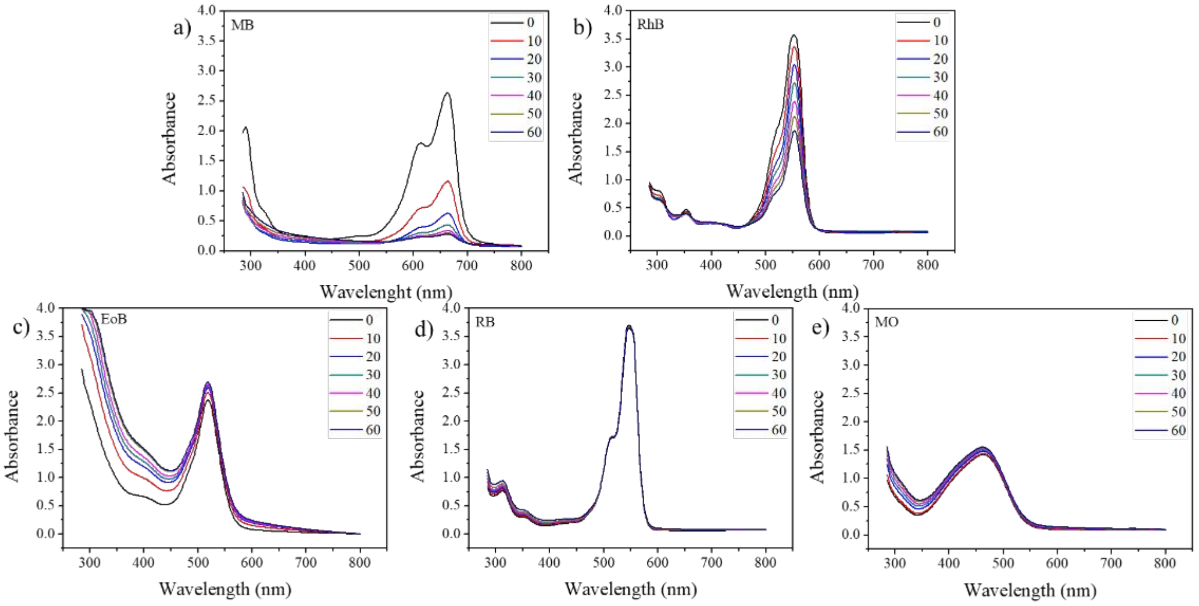

Figure S6. NR-Cat adsorption profiles for different dyes: a) methylene blue (MB), b) rhodamine B (RhB), c) eosin B (EoB), d) rose Bengal (RB) and e) methyl orange (MO).

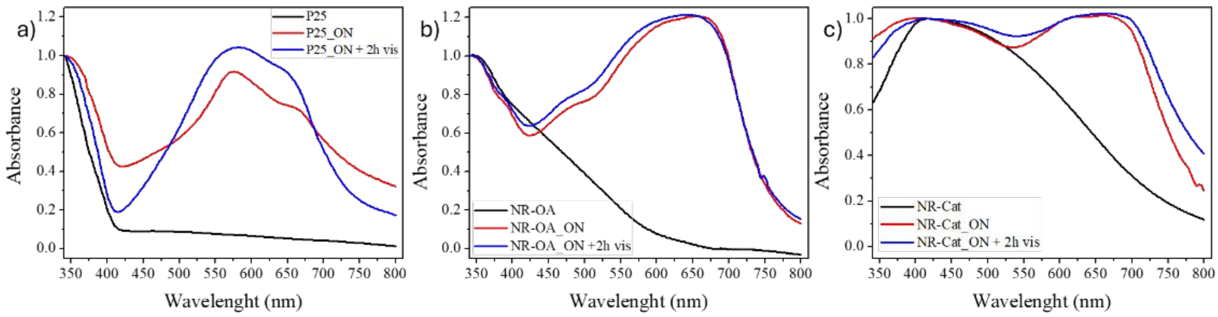

Figure S7. Diffuse reflectance spectroscopy (DRS) spectra of the pristine nanomaterial, the material after overnight contact with MB solution and subsequent 2h exposure to visible light for a) P25, b) NR-OA and c) NR-Cat.

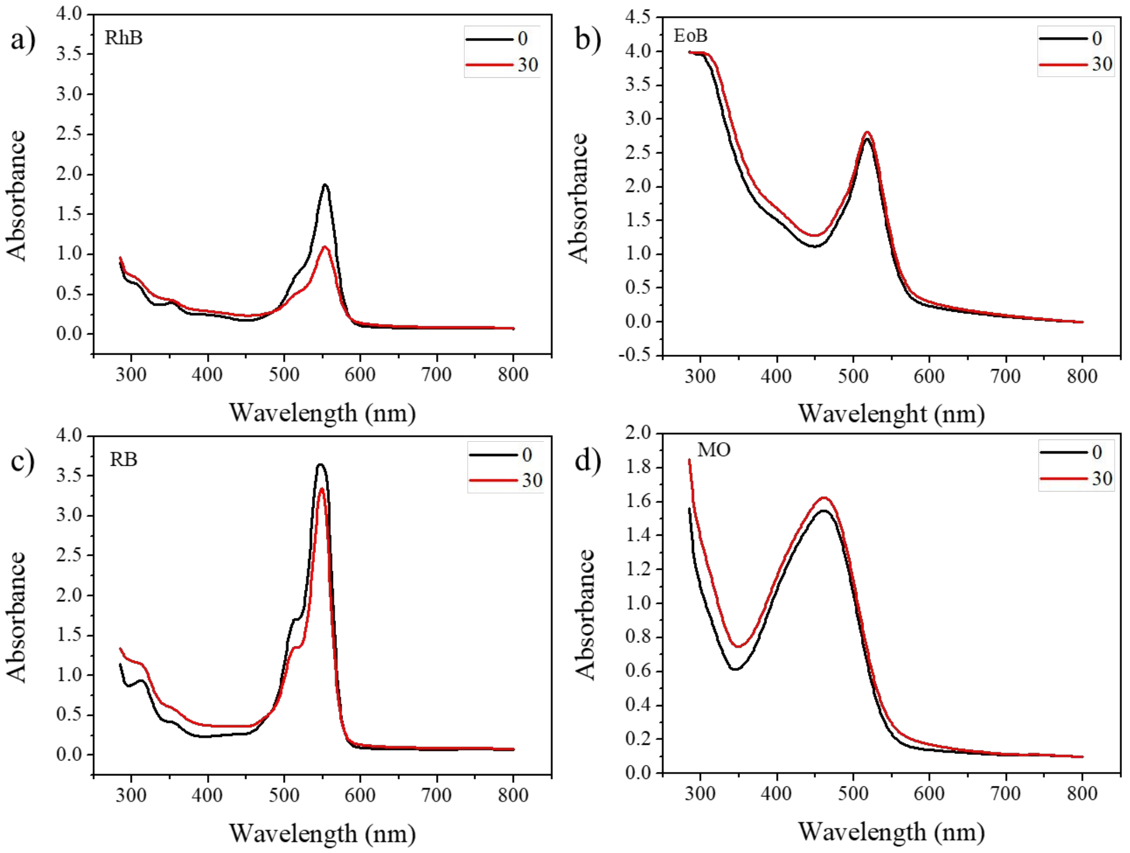

Figure S8. Absorbance spectra before and after 30 min exposure to light in presence of NR-Cat of different dyes solutions: a) RhB, b) EoB, c) RB, d) MO.
